# Computerized Memory Enhancement using NeuroFlex for Patients with Mild Cognitive Impairment: Results of a Pilot and Feasibility Study

**DOI:** 10.1002/alz70859_103032

**Published:** 2025-12-25

**Authors:** Alexander C Conley, S Shizuko Morimoto, Jennifer N Vega, John Patrick Begnoche, Nicole TP Nguyen, Soniya Patel, Roger A Altizer, Paul A Newhouse

**Affiliations:** ^1^ Center for Cognitive Medicine, Vanderbilt University Medical Center, Nashville, TN USA; ^2^ University of Utah, Salt Lake City, UT USA; ^3^ T.H. Chan School of Medicine, University of Massachusetts, Worchester, MA USA; ^4^ Center for Cognitive Medicine, Vanderbilt University Medical Center, Nashville, TN, USA, Nashville, TN USA; ^5^ Vanderbilt University Medical Center, Nashville, TN USA; ^6^ GRECC, VA, Tennessee Valley Healthcare System, Nashville, TN USA

## Abstract

**Background:**

The use of cognitive training as a therapeutic intervention for adults with Mild Cognitive Impairment (MCI) offers the potential for improved cognition without the adverse effects seen in many pharmacological trials. A novel cognitive enhancement strategy is neuroplasticity‐based computerized cognitive remediation (nCCR), which aims to boost cognitive performance by training clinically relevant cortical networks. In MCI patients, the goal is that by engaging networks that support memory function through nCCR, this will produce sustained improvements in functioning. The present study is a preliminary investigation into the feasibility and tolerability of an nCCR intervention called Neuroflex in adults with MCI.

**Method:**

This open‐label study required participants to complete 45‐hours of Neuroflex across six weeks. Training was delivered remotely through research tablet computers by study staff coaching over videoconferencing software. During and following the intervention, participants’ completed questionnaires and tasks that assessed potential changes in cognitive performance, which would indicate markers of functional engagement for future randomized trials.

**Result:**

The use of Neuroflex was well tolerated, as of the nineteen participants who were enrolled in treatment (11 female, mean age 73.3 ± 9 years), sixteen successfully completed training (completion rate: 84.2 %). Analyses of the open label data has shown improvements in self‐ and informant‐reported functioning, as well as in cognitive performance. Following NeuroFlex training, participants reported higher levels of perceived personal strengths on the Older Adults Self Report (*p* = 0.047, *d* = 0.54), and study partners reported lower levels of dementia symptoms on the Older Adult Behavior Checklist (*p* = 0.034, *d* = 0.58). Participants had improved working memory performance on the 2‐back task following training compared to baseline (*p* = 0.028, *d* = 0.63), and improved accuracy on a go/nogo task (*p* = 0.031, *d* =0.62).

**Conclusion:**

These results show that Neuroflex is feasible and well tolerated for use in patients with MCI. The benefits in cognitive performance indicate the potential for using Neuroflex as an augmentation to other therapeutics in adults with MCI and support the further investigation of Neuroflex with a randomized controlled trial.

